# Long Non-Coding RNAs in the Tumor Immune Microenvironment: Biological Properties and Therapeutic Potential

**DOI:** 10.3389/fimmu.2021.697083

**Published:** 2021-07-06

**Authors:** Ya-Nan Pi, Wen-Cai Qi, Bai-Rong Xia, Ge Lou, Wei-Lin Jin

**Affiliations:** ^1^ Department of Gynecology, Harbin Medical University Cancer Hospital, Harbin, China; ^2^ Department of Gynecology, The First Affiliated Hospital of USTC, Division of Life Sciences and Medicine, University of Science and Technology of China, Hefei, China; ^3^ Institute of Cancer Neuroscience, Medical Frontier Innovation Research Center, The First Hospital of Lanzhou University, The First Clinical Medical College of Lanzhou University, Lanzhou, China

**Keywords:** LncRNA, tumor microenvironment, immunosuppression, immune escape, therapeutic target

## Abstract

Cancer immunotherapy (CIT) is considered a revolutionary advance in the fight against cancer. The complexity of the immune microenvironment determines the success or failure of CIT. Long non-coding RNA (lncRNA) is an extremely versatile molecule that can interact with RNA, DNA, or proteins to promote or inhibit the expression of protein-coding genes. LncRNAs are expressed in many different types of immune cells and regulate both innate and adaptive immunity. Recent studies have shown that the discovery of lncRNAs provides a novel perspective for studying the regulation of the tumor immune microenvironment (TIME). Tumor cells and the associated microenvironment can change to escape recognition and elimination by the immune system. LncRNA induces the formation of an immunosuppressive microenvironment through related pathways, thereby controlling the escape of tumors from immune surveillance and promoting the development of metastasis and drug resistance. Using lncRNA as a therapeutic target provides a strategy for studying and improving the efficacy of immunotherapy.

## Introduction

Antitumor therapy is based on two fundamental principles: 1) direct killing of tumor cells or 2) regulation of the tumor microenvironment. To achieve the goal of tumor eradication, cancer biology cannot be understood based on the characteristics of tumor cells alone, but must also include the effect of the tumor microenvironment (TME) on the tumor (1). Therefore, there are some cases in which treatment methods that directly target tumor cells fail to achieve the expected efficacy in clinical application (1). Although Stephen Paget first proposed the “seed and soil” hypothesis in 1889, the subsequent renewed understanding of the TME has made it an important target for tumor research and therapy (2–4). The TME is a complex and dynamic network structure composed of tumor cells and the surrounding region (including tumor-associated immune cells, fibroblasts, vascular endothelial cells, adipocytes and extracellular matrix, as well as secreted cytokines and chemokines) (5). The immune microenvironment, hypoxic niche, metabolism microenvironment, acidic niche and innervated niche microenvironment, and other microenvironments are interconnected, which significantly contributes to the complexity and heterogeneity of the TME (6–12). The immune microenvironment is considered to be a critical specialized microenvironment that can reprogram cancer biology, and is closely related to cancer prognosis and response to treatment (7, 13).

The immune microenvironment is primarily composed of myeloid cells [i.e., macrophages, myeloid inhibitory cells (MDSCs), and neutrophils], lymphocytes [i.e., CD4+ T helper cells (Th), regulatory T cells (Tregs), CD8+ cytotoxic T cells (CTLs), B cells, natural killer (NK) cells, and dendritic cells (DCs)]. The composition and status of immune cells varies between different types of tumors and between patients with the same tumor (14). Both activated and suppressive immune phenotypes have been found in the TME based on the infiltration of immune cells (1). Moreover, high resolution single-cell RNA sequencing, flow cytometry, and immunoscore techniques have been applied in an effort to further understand the density and diversity of tumor-infiltrating immune cells (14–18). While these methods can help explain how immunotherapy-based strategies improve clinical outcomes, the therapeutic responses of immunotherapy-based strategies are limited to a small number of patients who have significantly improved patient-specific clinical outcomes (19, 20). In addition, reversing immunosuppressive strategies can improve the efficacy of immunotherapy (21). Immune escape and therapy resistance are the two major obstacles associated with radical tumor therapy, which are also primarily mediated by an immunosuppressive microenvironment (22, 23). Therefore, immune microenvironment reprogramming represents the key to improving the antitumor response and is a powerful target for CIT.

LncRNA is a type of non-coding RNA (ncRNA) longer than 200 nucleotides (24). LncRNAs have been found to be associated with multiple types of cancer [e.g., breast (25), lung (26), and liver (27) cancer], as well as resistance to chemotherapy and immunotherapy (28, 29). LncRNAs do not directly encode proteins involved in the innate or adaptive immune response; however, they can regulate the differentiation and function of immune cells (30). LncRNAs can also facilitate the escape of tumor cells from immune surveillance by promoting the formation of an immunosuppressive microenvironment and other mechanisms (31). For example, the lncRNA NKILA can induce the apoptosis of tumor-specific T cells so that they cannot penetrate the tumor (32). Recently, lncRNA has been considered a potential target for immunotherapy, and has attracted extensive attention in the field of cancer therapy research. This review primarily focuses on lncRNA-mediated reprogramming of the tumor immune microenvironment (TIME). In particular, we describe the mechanism by which lncRNA inhibits the generation of the microenvironment, inducing immune escape and immune checkpoints to promote resistance. Next, we summarize the potential application of lncRNA as a target for tumor immunotherapy.

## LncRNA: Molecular Features and Biological Mechanisms

In the human genome, approximately 93% of DNA can be transcribed into RNA, of which only 2% is protein-coding mRNA and the remaining 98% is termed non-coding RNA (33). LncRNAs lack protein-coding ability, can be spliced, capped, and/or polyadenylated, and are localized in the nucleus or cytoplasm (34). Based on their localization and the length between protein coding target mRNAs, lncRNAs can be roughly divided into intronic, intergenic, sense, antisense, bidirectional, and enhancer lncRNAs (35). Since the advent of the genomic era in the 2000s, significant progress has been made in understanding the biogenesis and function of different types of lncRNAs that are found ubiquitously across species (36, 37). The lncRNA is no longer considered to be “transcriptional noise”, but rather a highly efficient RNA factor (38, 39) that functions through epigenetic control and transcription, translation, RNA metabolism, and other mechanisms (40, 41). LncRNAs act as competing endogenous RNA (ceRNA) to competitively bind to miRNAs, thereby preventing miRNAs from binding to target mRNA (42–44). LncRNAs are directly involved in the epigenetic regulation of cancer by interacting with key histone modification enzymes, as well as chromatin modification, direct transcriptional regulation, and post-transcriptional functions (e.g., splicing, editing, localization, translation, and degradation) (45–47). Additionally, in gastric cancer, lncRNA SNHG17 has been shown to promote cancer progression by epigenetically silencing p15 and p57 (48). Recent studies have also suggested that *cis*-regulatory elements associated with the specific chromatin architecture are formed by epigenetic factors, endowing innate immune cells with specific phenotypes and unique functions by establishing cell-specific gene expression patterns (49). Moreover, lncRNAs can be used as immune modulators to regulate the immune response at the epigenetic level. Several studies have shown that lncRNAs are dysregulated in cancer and play a role in tumor proliferation, angiogenesis, apoptosis, and metastasis (50). In addition, lncRNAs are also closely related to the regulation of the TIME and antitumor immunity (50).

## LncRNA Regulation of the TIME: Focus on Immune Escape

LncRNAs play a regulatory role in the immune system. Immune regulation is achieved primarily through the processes of RNA/protein binding or RNA/DNA base pairing, and both lncRNA and mRNA use a common promoter region to conduct bidirectional transcription (51, 52). In addition, lncRNAs can regulate the immune response through several different pathways, including NF-κB/MAPK and JAK/STAT (53). MYC-regulated NEAT1 was found to promote diffuse large B cell lymphoma (DLBCL) proliferation *via* the miR-34b-5p-GLI1 pathway (54). It has also been reported that some immune-related lncRNAs control the differentiation, development, and effector function of these cells (55). Moreover, lncRNA can mediate the activation and inhibition of immune response genes. In a breakthrough study, lncRNA-DC was found to be expressed only in human DCs, directly bind to STAT3 in the cytoplasm, and promote STAT3 phosphorylation on tyrosine-705 by preventing the binding and dephosphorylation of STAT3 to SHP1. An Lnc-DC knockout was demonstrated to impair DC differentiation in human monocytes *in vitro* and mouse bone marrow cells *in vivo*, which decreased the ability of DCs to stimulate T cell activation (56). These results indicate that lncRNAs are key immunomodulators.

**Figure 1:**
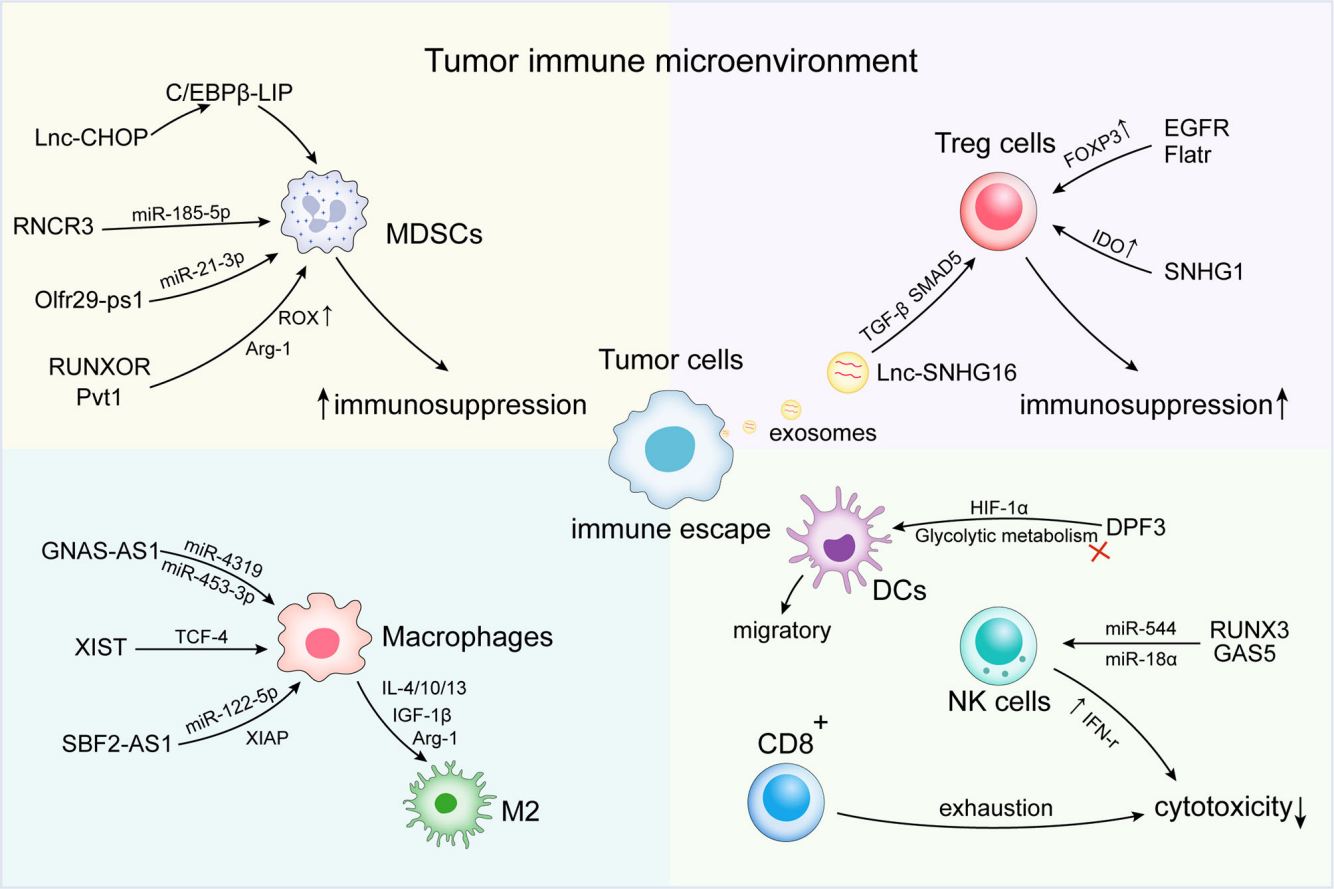
Long non-coding RNAs (lncRNAs) regulate immune escape in the tumor immune microenvironment (TIME). At the microenvironmental level, lncRNAs are involved in mediating and controlling various immune and cancer cell interactions, promoting the activation of immunosuppressive cells and factors [e.g., myeloid-derived suppressor cells (MDSCs) and tumor-associated macrophage (TAMs) subsets]. Abnormal antitumor immune cells [e.g., dendritic cell (DC), natural killer (NK) cells, and T cells] and regulatory cells T cells (Tregs) induce the formation of an immunosuppressive microenvironment, thus contributing to the immune escape of tumor cells.

Tumor cells can evade immune recognition and elimination by changing their phenotype or the microenvironment (57). The activation of immunosuppressive cells and factors [e.g., MDSCs, tumor-associated macrophage (TAMs) subsets], abnormal antitumor immune cells (e.g., DC, NK, and T cells), and Tregs represent important features of the microenvironment that promote tumor immune escape (58, 59). At the microenvironmental level, lncRNAs are involved in mediating and controlling various immune and cancer cell interactions and other important mechanisms of the immune response (**Table 1**). Various studies have confirmed that lncRNAs induce the formation of an immunosuppressive microenvironment through related pathways, thereby contributing to tumor escape of immune surveillance, as well as the development of metastasis and drug resistance (**Figure 1**).

**Table 1:** Summary of evidence for the role of lncRNA in the tumor immune microenvironment.

LncRNA	Cancer type	Related immune cell	Involved Molecules or pathways	Mechanisms	Ref
lnc-CHOP	MM, LLC, BC	MDSCs	CHOP	Promotes the activation of C/EBPβ and upregulates the expression of arginase-1, NO synthase 2, NADPH oxidase 2, and cyclooxygenase-2, which are related to the immunosuppressive function of MDSCs in inflammatory and tumor environments.	(60)
RNCR3	–	MDSCs	mir-185-5p	RNCR3/miR-185-5p/Chop autologously strengthening network promotes MDSC differentiation and suppressive functions in response to extracellular inflammatory and tumor-associated signals.	(61)
Olfr29-ps1	MM	MDSCs	miR-214-3p	Olfr29-ps1 may regulate the differentiation and function of MDSCs through a m6A-modified Olfr29-ps1/miR-214-3p/MyD88 regulatory network.	(62)
Pvt1	LLC	MDSCs	Arg1 and ROS	Enhances G-MDSC-mediated immunosuppression and inhibits the antitumor T cell response.	(63)
MALAT1	LC	MDSCs	Arg1	Negatively regulates MDSCs.	(64)
HOTAIRM1	LC	MDSCs	HOXA1-miR124	HOTAIRM1 enhances the expression of HOXA1 in MDSCs and high levels of HOXA1, the target gene of HOTAIRM1, delays tumor progression and enhances the antitumor immune response by downregulating the immunosuppression of MDSCs.	(65)
RUNXOR	LC	MDSCs	Arg1	RUNXOR recruits EZH2 and RUNX1 to epigenetically regulate the RUNX1 gene in AML cells.	(66)
lnc-C/EBPβ	LCC, CC	MDSCs	Arg-1, CYBB (NOX2), NOS2, ptgs2(COX2)	Controls the immune-suppressive function and differentiation of MDSCs.	(67, 68)
lnc-EGFR	HCC	Tregs	EGFR, AP-1/NF-AT1	Stimulates Treg differentiation, suppresses CTL activity, and promotes HCC growth in an EGFR dependent manner.	(69)
SNHG1	BC	Tregs	miR-448/IDO	Accelerates the differentiation of Treg cells and promotes the immune escape of cancer by regulating the miR-448/IDO axis.	(30)
Flicr	–	Tregs	FoxP3	Escape from dominant Treg control during infection or cancer, at the cost of heightened autoimmunity.	(70)
Flatr	–	Tregs	FoxP3	Flatr promotes the expression of FOXP3 and enhances the immunosuppressive function of Tregs.	(71)
SNHG16	BC	Tregs	miR-16-5p, TGF-β1/SMAD5	Breast cancer-derived exosomes transmit SNHG16 to induce CD73+ γδ1 Treg cells by activating the TGF-β1/SMAD5 pathway.	(72)
POU3F3	GC	Tregs	TGF-β/SMAD2/3	Promotes the distribution of Tregs among peripheral blood T cells, increases cell proliferation by recruiting TGF-β, as well as activating the TGF-β signaling pathway.	(73)
RP11-323N12.5	GC	Tregs	YAP/TAZ/TEAD Hippo signaling	Promotes Treg cell differentiation by enhancing YAP1 transcription in T cells.	(74)
FENDRR	HCC	Tregs	miR-423-5p/GADD45B	Inhibits Treg-mediated immune escape of tumor cells through upregulating GADD45B by sponging miR-423-5p.	(75)
GNAS-AS1	NSCLC, BC	Macrophage	miR-4319, miR-433-3p, p53	Promotes M2 polarization of macrophages and NSCLC cell progression *via* directly inhibiting miR-4319. GNAS-AS1/miR-433-3p/GATA3 axis promotes the proliferation and metastasis of ER+ breast cancer cells by accelerating M2 macrophage polarization.	(76, 77)
XIST	LC	Macrophage	TCF-4	TCF-4 regulates lncRNA XIST in M2 polarization and provides novel insight into TAM regulation.	(78)
NIFK-AS1N	EC	Macrophage	NIFK-AS1/miR-146a/NOTCH1 axis	NIFK-AS1 inhibits the M2-like polarization of macrophages *via* targeting miR-146a, thereby reducing the estrogen-induced proliferation, migration, and invasion of endometrial cancer cells.	(79)
COX-2	HCC	Macrophage	IL-12, iNOS, and TFN-alpha (M1), Arg1, IL-10, and Fizz-1(M2)	Inhibits HCC immune evasion and tumor growth by inhibiting the polarization of M2 macrophages.	(80)
SBF2-AS1	PC	Macrophage	miR-122-5p/XIAP	lncRNA SBF2-AS1 in M2 macrophage-derived exosomes increases miR-122-5p expression to restrain XIAP expression, which further inhibits PC progression.	(81)
CCAT1	PC	Macrophage	miR-148a/PKCζ axis	Inhibits M2 polarization by down-regulating miR-148a.	(82)
Lnc-P21	BC	Macrophage	miR-1303	Promotes M2 Polarization in the tumor microenvironment, which might be caused by MDM2 eliciting proteasome-dependent p53. TAMs with an lincRNA-p21 knockdown induced cancer cell apoptosis, and inhibited tumor cell migration and invasion.	(83)
BCRT1	BC	Macrophage	miR-433-3p sponging, IL-10 and Arg1	LncRNA BCRT1 competitively binds with miR-1303 to prevent the degradation of its target gene PTBP3, which acts as a tumor-promoter in breast cancer. LncRNA BCRT1 overexpression could promote M2 polarization of macrophages, mediated by exosomes.	(84)
LINC00662	HCC	Macrophage	Wnt/β-catenin	LINC00662 activates Wnt/β-catenin signaling in macrophages in a paracrine manner and further promotes M2 macrophage polarization.	(85)
MALAT1	HCC	Macrophage	miR-140, VEGF-A	MALAT1-mediated FGF2 protein secretion from TAMs inhibits inflammatory cytokine release, promotes proliferation, migration, and invasion; the interaction between MALAT1 and miR-140 regulates angiogenesis and immunosuppressive properties.	(86, 87)
TUC339	HCC	Macrophage	IL-1 β, TNFα	TUC339 in macrophages diminishes the expression of M(IL-4) markers upon IL-4 treatment while overexpression of TUC339 in macrophages enhances M(IL-4) markers upon IFN-γ + LPS treatment, suggesting a critical function of TUC339 in the regulation of macrophage M1/M2 polarization.	(88, 89)
RPPH1	CRC	Macrophage	TUBB3	CRC cell-derived exosomes transport RPPH1 into macrophages which mediate macrophage M2 polarization, which in turn, promotes the metastasis and proliferation of CRC cells.	(90)
MM2P	OS	Macrophage	STAT6	Manipulating lncRNA-MM2P in macrophages impairs macrophage-mediated promotion of tumorigenesis, tumor growth in *vivo*, and tumor angiogenesis.	(91)
RP11-361F15.2	OS	Macrophage	miR-30c-5p, CPEB4	RP11-361F15.2 promotes CPEB4-mediated tumorigenesis and M2-like polarization of TAMs through miR-30c-5p in OS. RP11-361F15.2 also acts as a competitive endogenous RNA (ceRNA) against miR-30c-5p, thereby binding and activating CPEB4.	(92)
ANCR	GC	Macrophage	FOXO1	LncRNA ANCR in macrophages reduces the concentration of M1 macrophage marker molecules, IL-1β and IL-6, in the supernatant and inhibited M1 polarization of macrophages.	(93)
XIST	LC	Macrophage	IL-4, TCF-4	Promotes M2 polarization.	(94)
CASC2	GM	Macrophage	miR-338-3P	CASC2c and miR-388-3p bind to FX and commonly inhibit its expression and secretion. CASC2c suppresses M2 macrophage polarization, and alters the GBM microenvironment.	(95)
SNHG20	HCC	Macrophage	STAT6	SNHG20 may facilitate the progression of NALFD to HCC *via* inducing liver KC M2 polarization *via* STAT6 activation.	(96)
LIFR-AS1	Os	Macrophage	miR-29a/NFIA	Macrophage-derived exosomal lncRNA LIFR-AS1 can promote osteosarcoma cell proliferation, invasion, and restrain apoptosis *via the* miR-29a/NFIA axis.	(97)
Lnc-Dpf3	–	DCs	HIF-1α	DC-specific lnc-Dpf3 deficiency increases CCR7-mediated DC migration, leading to exaggerated adaptive immune responses and inflammatory injuries.	(98)
Lnc-DC	–	DCs	STAT3, TLR9, TIMP, MMP	Lnc-DC promotes DC maturation and inhibits trophoblast invasion without the involvement of CD4+ T cells. Lnc-DC controls the immune response by reducing the concentration of TNF-α, IL-6, IL-12, and IFN-γ, as well as increasing the concentration of IL-1β secreted by dendritic cells.	(99, 100)
NEAT1	–	DCs	miR-3076-3p/NLRP3	NEAT1 induces a tolerogenic phenotype in DCs.	(101)
HOTAIRM1	–	DCs	miR-3960/HOXA1	Regulates DC differentiation by competitively binding to endogenous miR-3960.	(102)
MALAT-1	CC	DCs	SNAIL	Blocking MALAT-1 significantly decreases the TADC-conditioned medium and CCL5-mediated migration and invasion by decreasing Snail.	(103)
Lnc-CD56	–	NKs	CD56	Positive regulator of CD56.	(104)
GAS5	HCC, GC	NKs	miR-544/RUNX3, miR-18a	LncRNA GAS5 overexpression enhances the killing effect of NK cell on liver cancer through regulating miR-544/RUNX3. Promotes NK cell cytotoxicity against gastric cancer by regulating miR-18a.	(105, 106)
IFNG-AS1	–	NKs	IFNG	Enhances IFN-γ in human natural killer cells.	(107)
lincEPHA6-1	LC	NKs	miR-4485-5p/NKp46	linc-EPHA6-1 acts as a competing endogenous RNA (ceRNA) for hsa-miR-4485-5p, which subsequently up-regulates natural cytotoxicity receptor (NKp46) expression.	(108)
lnc- TIM-3	HCC	CD8+ T	TIM-3	Lnc-Tim interacts with Tim-3 to release Bat3 and induces CD8+ T cell exhaustion, promoting HCC immune evasion.	(109)
NEAT1	HCC	CD8+ T	miR-155, TIM-3	Suppression of NEAT1 restrains CD8+ T cell apoptosis and enhances the cytolysis activity against HCC *via* modulating the miR-155/Tim-3 pathway.	(110)
lnc-sox5	CC	CD8+ T	IDO1	Suppresses the infiltration and cytotoxicity of CD8+ T cells and promotes tumorigenesis.	(111)

MM, melanoma; LLC, Lewis lung carcinoma; BC, breast cancer; LC, lung cancer; CC: colon cancer; HCC, hepatocellular carcinoma; GC: gastric cancer; NSCLC, non-small cell carcinoma lung cancer; EC, endometrial cancer; PC, prostate cancer; CRC, colorectal cancer; OS, osteosarcoma; GM, glioblastoma multiforme; CHOP, C/EBPβ homologous protein; Arg1, arginase-1; ROS: reactive oxygen species; EZH2, enhancer of zeste homolog 2; RUNX1, runt-related transcription factor 1; EGFR, epidermal growth factor receptor; IDO: indoleamine 2,3-dioxygenase; FoxP3, forkhead box protein 3; GADD45B, DNA-damage-inducible beta protein; TCF-4, T-cell-specific transcription factor 4; XIAP, X-linked inhibitor of apoptosis protein; PKCζ, protein kinase C zeta; VEGF: vascular endothelial growth factor; TUBB3, β-III tubulin; CPEB4, cytoplasmic polyadenylation element binding protein 4; HIF-1α, hypoxia inducible factor-1 α; STAT, signal transducer and activator of transcription; TLR9, Toll-like receptor 9; TIMP, tissue inhibitor of metalloproteinase; NLRP3, NOD-like receptor pyrin domain-containing 3; IFNG, interferon gamma; TIM-3, T cell immunoglobulin and mucin-domain containing-3.

### LncRNA Regulation of MDSCs

LncRNAs can inhibit the immune response by regulating the activity of immunosuppressive cells. Under pathological conditions, extramedullary bone marrow generates MDSCs (112). MDSCs play a central role in cancer progression by mediating immunosuppression in the TME through a variety of mechanisms, including the production of inducible nitric oxide synthase (iNOS), arginase-1 (Arg1), oxygen free radicals (ROS), and nitric oxide (NO) (113, 114). Recent studies suggest that lncRNAs play an important role in the immunosuppressive functions of MDSCs. In particular, lnc-CHOP and RNCR3 can positively regulate the growth and inhibitory function of MDSCs (60, 61). Moreover, lnc-chop may interact with CHOP and the C/EBPB isoform, LIP, to encourage C/EBPB activation. C/EBPB is associated with the differentiation of MDSCs. Therefore, lnc-chop may affect the differentiation of MDSCs and activate the expression of immunosuppressive genes. Furthermore, the combination of lnc-chop and CHOP in MDSCs may have important significance for the control of tumor growth, since increased CHOP expression in tumor-associated MDSCs has been observed in a variety of tumor models (115). Similarly, the expression of the lncRNA, RNCR3, in MDSCs is upregulated by both inflammatory and tumor-associated factors. In addition, an RNCR3 knockout was found to result in suppressed MDSC differentiation and function both *in vitro* and *in vivo* (61). Another study found that the lncRNA, pseudogene Olfr29-ps1, is expressed in MDSCs. Pseudogene Olfr29-ps1 has been shown to regulate the differentiation and immunosuppressive function of MDSCs *via* the N6-methyladenosine (M6A) modified Olfr29-ps1/miR-214-3p/MyD88 regulatory network (62, 116). The lncRNAs, Pvt1, MALAT1, HOTAIRM1, RUNXOR, and others have also strongly confirmed the regulatory effect of lncRNA on MDSC activity (63–66).

### LncRNA Regulation of Tregs

Tregs are an immunosuppressive subset of CD4+ T cells (117). The depletion of CD4+CD25+ regulatory T cells promotes a tumor-specific immune response in the pancreas cancer-bearing mice (118). Tumor-infiltrating Tregs may also interfere with host antitumor responses by inhibiting tumor-specific immune effector cells. Multiple studies have shown that lncRNAs [e.g., lnc epidermal growth factor receptor (lnc-EGFR), lncRNA SNHG1, Flicr, and Flatr] can regulate the biological function of Tregs (30, 69–71). The upregulation of lnc-EGFR in Tregs was positively correlated with tumor size and EGFR/Foxp3 expression. Lnc-EGFR functions by activating the downstream AP-1/NF-AT1 axis and inducing EGFR expression. Moreover, lnc-EGFR has also been shown to stimulate Treg differentiation, inhibit CTL activity, and promote hepatocellular carcinoma (HCC) growth (69).

The lncRNA, Flatr, is a part of the upstream cascade that leads to enhanced differentiation, FOXP3 expression, and immunosuppressive function in Tregs (71). Breast cancer cells promote the expression of SMAD5 in γδT cells through the transfer of the lncRNA, SNHG16, in exosomes, which functions as a ceRNA through miR-16-5p, thereby enhancing the TGF-β1/Smad5 pathway and upregulating CD73 expression (72). The study by Pei et al. showed that interference with SNHG1 promoted miR-448 expression, reduced the level of indoleamine 2,3-dioxygenase (IDO), and inhibited Treg differentiation, thereby impeding tumor immune escape (30).

### LncRNA Effects Macrophage Differentiation in Immune Escape

In the immune microenvironment, macrophages are classified as proinflammatory, antitumorigenic M1, and anti-inflammatory protumorigenic M2 phenotypes (119). TAMs function by directly or indirectly inhibiting effector T cells (120). Multiple studies have shown that lncRNA can affect the immune escape of tumor cells by regulating M2 macrophage polarization. LncRNA GNAS-AS1 expression is significantly enhanced in TAM non-small cell lung cancer (NSCLC) cell lines and clinical tumor tissues in lung cancer, and is negatively correlated to the overall survival of NSCLC patients. Moreover, lncRNA GNAS-AS1 promotes tumor progression in NSCLC by altering macrophage polarization through the GNAS-AS1/MIR4319/NECAB3 axis (76). LncRNA-XIST is regulated by TCF-4, which also plays a role in promoting M2 macrophage polarization (78). However, some lncRNAs can negatively regulate TAM M2 polarization. In endometrial cancer, NIFK-AS1 inhibited the M2-like polarization of macrophages by targeting miR-146a, thereby reducing the proliferation, migration, and invasion of estrogen-induced endometrial cancer cells (79). Zhou et al. co-incubated a mouse liver cell line (HEPAL6 cells) and a liver cancer cell line (HepG2 cells) with M1 or M2 macrophages, and found that lncRNA COX-2 expression was higher in M1 macrophages than in M2 macrophages. LncRNA COX-2 inhibits HCC immune escape and tumor growth by inhibiting M2 macrophage polarization (80). In pancreatic cancer, studies have shown that the blocking Sbf2-AS1 in M2 macrophage-derived exosomes inhibited XIAP expression through the negative regulation of miR-122-5p, and played a role in reducing the oncogenic ability of tumor cells (81). In the report by Zhang et al., RNA sequencing and other methods were used to identify differentially expressed miRNAs and lncRNAs in MΦ-CM co-cultured osteosarcoma cells and the corresponding control group, which confirmed that lncRNA LIFR-AS1 was upregulated in MΦ-CM co-cultured osteosarcoma cells (97). In addition, LIFR-AS1 can be transmitted from macrophages to osteosarcoma cells *via* exosomes, and promote tumor progression *via* spongy transfection of miR-29a (97). These findings show that abnormally expressed lncRNAs can be used as potential biological targets for cancer therapy.

### LncRNA Modulation of Antitumor Immune Cells

DCs are associated with overall survival in cancer patients, reflecting the unique ability of humans to initiate CD8+ T cell responses (121). However, the TIME often interferes with the normal function of DCs to avoid immune surveillance (122, 123). LncRNAs can regulate DC infiltration, differentiation, and metabolism, as well as influence other immune cells, including T cells, to modify the local immune environment. Lnc-DC was found to promote DC maturation and inhibit trophoblast invasion without the involvement of CD4+ T cells. In addition, lnc-DC controlled the immune response by reducing the concentration of TNF-α, IL-6, IL-12, and IFN-γ secretion, as well as increasing IL-1β production by DCs (99, 100). Lnc-DPF3 inhibits DC migration by directly binding to HIF1A and inhibiting HIF1A activity *via* the HRE motif to suppress glycolysis (98).

The first line of immune defense includes NK cells, which are cytotoxic immune cells that can directly kill cancer cells (124). Recently, Zhang et al. measured the expression profile of lncRNAs in human primary lymphocytes, and found that NK-specific lncRNAs are closely associated with the differentiation and function of NK cells. The expression of the NK-specific lncRNA, lnc-CD56, was found to be a positive regulator of CD56 (104). In addition, the lncRNA, GAS5, could regulate the killing effect of NK cells in several types of cancer (105, 106). These results demonstrate the importance of lncRNAs in NK cell function and the antitumor immune response. LncRNA can regulate the function of CD8+ T cells in the TME through a variety of mechanisms to alter the immune response. LNC-TIM3 was found to be upregulated in tumor-infiltrating CD8 T cells from HCC patients and negatively correlated with the level of IFN-γ and IL-2 production. LNC-TIM3 specifically binds to Tim-3 and blocks its interaction with BAT3, thereby inhibiting the downstream LCK/NFAT1/AP-1 signaling pathway, and plays a key role in promoting CD8 T suppression (109). In another study, both NEAT1 and TIM-3 expression were upregulated in the PBMCs of liver cancer patients compared with healthy subjects. The downregulation of NEAT1 can inhibit the apoptosis of CD8+ T cells through the miR-155/Tim-3 pathway, enhance cell lysis activity, and inhibit tumor growth in mice with HCC (110).

## LncRNA Impacts Resistance to Immune Checkpoint Therapy

Immunotherapy, primarily represented by PD-1/PD-L1 inhibitors, has made substantial breakthroughs for the treatment of multi-solid tumors (125–127). Thus, immunotherapy has become a popular form of cancer treatment. However, after experiencing an initial response to immune checkpoint inhibitor (ICIS) therapy, most patients develop secondary resistance. The mechanism by which secondary resistance develops remains largely uncertain (128, 129). ICIS therapy functions by relieving the immunosuppression of tumor cells or the associated microenvironment. Several factors can impact the efficacy of ICIS, including antigen presentation, tumor mutation burden, and T cell infiltration (128, 130).

The mechanism of immune escape is dominated by the formation of an immunosuppressive microenvironment, which can be regulated by lncRNAs. Some lncRNAs also promote the generation of drug resistance through the PD-1/PD-L1 axis and the presentation of inhibitory antigens. For example, the lncRNA, MALAT1, can regulate tumor immunity by indirectly upregulating the expression of PD-L1 through miR-195 and miR-200a-3 (131, 132). In addition, the SNHG14/miR-5590-3p/ZEB1 positive feedback loop was found to promote the progression and immune escape of DLBCL by regulating the PD-1/PD-L1 checkpoint, suggesting that targeting SNHG14 is a potential method of improving the efficacy of DLBCL immunotherapy (133). More importantly, silencing LINC00473 resulted in increased expression of Bcl-2 X-related proteins (Burlington), interferon (IFN)-γ, and IL-4, but reduced the expression of B cell lymphoma-2 (Bcl-2), matrix metalloproteinase (MMP-2), MMP-9, and IL-10, thereby inducing enhanced apoptosis and inhibiting proliferation of DLBCL. In addition, silencing LINC00473 or elevating miR-195-5p was found to increase the number of activated CD8+ T cells (134). In contrast, NKX2-1-AS1 has been shown to aid in inhibiting immune escape by negatively regulating PD-L1 (135). LncRNA can also regulate antigen presentation, as Link-A has been shown to inactivate tumor suppressor pathways and downregulate antigen presentation through inactivating the PKA pathway. Therapy with Link-A locked nucleic acid or a GPCR antagonist has been found stabilize the PLC components, Rb and p53, and sensitize breast tumors to immune checkpoint blockers. Elevated Link-A levels were also confirmed in patients with programmed cell death protein 1 (PD-1)-blocking triple-negative breast cancer (TNBC) (31). Therefore, the regulation of lncRNA plays a key role in resistance to ICIS therapy.

## Potential Therapeutic Approaches of lncRNA as Important Targets

From a clinical perspective, lncRNA-mediated regulation of the immune microenvironment represents a highly promising target for immunotherapy. There are many therapeutic strategies targeting lncRNAs, including small molecule inhibitors, antisense oligonucleotides (ASOs), RNA interference (RNAi) technology, and Clustered Regularly Interspaced Short Palindromic Repeats (CRISPR)/Cas9 genome editing (136). Small molecule inhibitors mainly bind to the higher structural regions of lncRNAs that are similar to protein targets (137). Screening and identification of small molecule compounds that may inhibit RNA can be achieved by high-throughput sequencing. ASO belongs to a class of drugs that bind to the lncRNA transcriptome *via* base pairing (138). Gapmer was developed based on this mechanism, and uses RNA nucleotides with extra covalent bonds to 2 ‘-O and 4’ -C nucleotide rings to specifically bind to RNA targets and recruit the RNA-H enzyme to induce target degradation (139). RNAi is a biological process of inducing a specific gene knockout by neutralizing targets with exogenous double-stranded RNA, including both short interfering RNAs (siRNAs; with high specificity and short effects) and short hairpin RNAs (shRNAs; with long-lasting and stable effects) (140). Moreover, the CRISPR/Cas9 system can be used to silence or knock-out lncRNA-expressing loci (141). After the CRISPR/CAS system enters the cell, gRNAs guide the CAS enzyme to locate specific DNA sequences on PAM complementary to the gRNA, after which the CAS enzyme will cut the DNA double strand, changing it or inducing a mutation through a frameshift, which finally leads to the silencing of the edited gene (142). Off-target effects represent the main difficulties associated with CRISPR/Cas9 gene therapy.

The key to treatment is to optimize target delivery. As therapeutic carriers, nanomaterials and exosomes can protect against drug degradation or aggregation and are associated with good targeting. As such, the safe and efficient intracellular delivery of CRISPR/Cas9 is critical for effective therapeutic genome editing. The study by He et al. showed that the use of epithelial cell-derived microvesicles (MVS) as a carrier to deliver CRISPR/Cas9 components to cancer cells showed strong anticancer effects against xenograft tumors, and this may become a safe CRISPR/Cas9 delivery platform for cancer patients (143). Furthermore, the application of nanotechnology can maximize the advantages of using lncRNA in combination with immunotherapy.

### Nanoparticles

Over the past decade, the development of nanoparticle platforms has yielded promising prospects for their application in RNA therapy and cancer immunotherapy (144). Nanoparticles are granular dispersions or solid particles ranging in size from 10 nm to 1000 nm. Therapeutics can be delivered using nanoparticles to achieve enhanced permeability and retention (EPR). Typical nanoparticles include liposomes, polymer nanoparticles (NPs), inorganic NPs, and exosomes (145–149). Nanocarriers also typically exhibit good biocompatibility and stability. Moreover, nanoparticles can be customized *via* unique physical properties (e.g., dimensional charge and surface chemistry), to enable specific tissue or tumor targeting. On their own, nano pharmaceuticals can enhance cellular interactions, stimulate the immune system, and sustain an antitumor response (150). Therefore, the use of nanoparticles as a lncRNA-targeted therapy carrier combined with immunotherapy represents a multi-effect strategy. Gong et al. successfully constructed MALAT1-specific ASO and nucleo-targeted Tat peptide synergized Au nanoparticles (i.e., ASO-Au-Tat NPS), which could stabilize fragile ASOs, enhance nuclear internalization, and demonstrate good biocompatibility. Following treatment with ASO-Au-Tat NPS, the level of MALAT1 expression in A549 lung cancer cells was significantly reduced. In addition, ASO-Au-Tat NPS has been found to significantly reduce the formation of metastatic tumor nodules *in vivo* (151). Another study demonstrated that RGD-peg-ECO/siDANCR nanoparticle treatment of MDA-MB-231 cells and BT549, siRNA could be effectively passed to the cell, and continue to silence targeted nanoparticles. In addition, combined treatment significantly reduced TNBC cell survival, proliferation and tumor globular form of migration (152). In a recent study, researchers designed a novel type of polymer nanoparticle, which simultaneously targeted T cell immunoreceptor with Ig and ITIM domains (TIGIT)/polio virus receptors (PVRs), T cell immune receptors, and long non-coding RNA antisense non-coding RNA in the INK4 locus (lncRNA ANRIL) to suppress liver cancer. DTTP/3NP/siANRIL have a good antitumor effect against liver cancer, and inhibition of miR-203a and its downstream gene expression increases the percentage of NK cells and T cells (153). Nanoparticle-based delivery systems not only deliver high-dose therapeutic payloads to target cells, but also exhibit the same regulatory function in immunotherapy with RNA therapy. At the same time, combining lncRNA-mediated nanotherapy with existing immunotherapy provides an opportunity to improve the efficacy of cancer treatment. However, relatively few studies have investigated the use of this delivery method, and it will take some time before this application can be used in the clinic.

### Exosomes

Exosomes are extracellular nanovesicles (30–150 nm in diameter) of endocytic origin that are secreted by most mammalian cell types. Exosomes are present in a wide range of bodily fluids (154). Moreover, exosomes are now recognized as important intercellular signaling messengers that encapsulate and transfer versatile molecular cargo to recipient cells. Exosomes naturally possess sophisticated specificity and are capable of passing through most biological barriers *in vivo* (155). Exosomes engineered to deliver specific small interfering RNA (siRNA) payloads can be protected from degradation by blood-derived ribonuclease (156). In addition, the surfaces of exosomes can be designed with required ligands to increase targeting efficiency (157). For example, exosomes can effectively deliver microRNAs (miRNAs) to breast cancer cells expressing epidermal growth factor receptor (EGFR) (158). Furthermore, exosomes themselves can regulate innate and acquired immunity, as well as the TME (119).

Exosomes have the unique advantages of improving cancer therapeutic indicators. They can also be engineered into therapeutic exosomes that improve the efficiency and targeting ability of antitumor drugs. Exosomes with siRNA targeting KRAS^G12D^ were shown to reduce KRAS GTPase activity and the downstream activation of RAF-MEK-ERK or PI3K-AKT-mTOR signaling, inhibit cancer cell proliferation, and increase pancreatic cancer cell apoptosis (159, 160). Recently, a trial using exosome vectors as a means of siRNA delivery was conducted in breast cancer cells. These exosomes were able to specifically bind to HER2/Neu and were capable of delivering siRNA molecules against the TPD52 gene into a SKBR3 cell line, which downregulated TPD52 gene expression by up to 70% (161). In addition, exosomal AFAP1-AS1 was found to induce trastuzumab resistance through associating with AUF1 and promoting ERBB2 translation (162).

Since lncRNAs play an important role in tumor immune escape and immunotherapy resistance, targeted lncRNA drugs combined with immunotherapy may provide an effective strategy for the treatment of cancer. For example, link-A may represent a potential therapeutic target for increasing ICIS sensitivity (31). Moreover, NKILA silencing in metastatic tumor infiltrating lymphocytes and CAR T cells can overcome tumor immune escape and improve the efficacy of adoptive T cell therapy in cancer treatment (32). Thus, nanoparticle or exosome-loaded lncRNA targeted therapy combined with immunotherapy has broad applicability in the field of cancer therapy.

## Challenges and Future Perspectives

This article mainly reviews the reprogramming of the TIME mediated by lncRNAs. The role and mechanism of lncRNA in the formation of an inhibitory microenvironment and in inducing tumor cells to escape immune surveillance have been described in detail. The TME is highly complex. Although the importance of lncRNA in the regulation of the TIME has been demonstrated, a clear mechanism remains to be elucidated. Next, we discussed relevant strategies for targeting lncRNA therapy, which can improve therapeutic efficacy and accelerate clinical application by optimizing the targeted delivery of vectors. LncRNA is both a potential therapeutic target for cancer, as well as a predictor of the survival and treatment response. Tu et al. found that MSC derivatives induced the expression of LINC01119 in adjacent TNBC cells and accelerated the growth of cancer cells *in vitro*. LINC01119 is a strong prognostic indicator for poor prognosis in patients with TNBC (163). Additionally, lncRNA-based therapy is a promising approach in the field of cancer immunotherapy (164). In a recent cohort study, overall survival (OS) with immunofunctional lncRNA features and high CTL infiltration benefited the most. At the same time, a multiomics panel based on lncRNA score has been designed as a useful biomarker for cancer immunotherapy (165). Another study demonstrated that lncRNA miR155 was closely associated with the OS of different tumor types, immune cell infiltration, and immune checkpoint molecule expression, and also provided great value for predicting the efficacy of immune checkpoint inhibitor therapy (166). Thus, lncRNA-based immune subtypes are associated with survival and response to cancer immunotherapy.

Recent findings offer novel insight into lncRNA-based cancer treatment and draw attention to areas that require further research; however, there are some problems that remain to be solved. The application of lncRNA as a therapeutic target is associated with several challenges, the most important of which is the method by which specific molecules can be delivered to target cells. Next, problems with molecular delivery and off-target effects may cause safety concerns during treatment. The application of nanoparticles and exosome carriers are the key to solving these problems, and these platforms have good targeting. However, there are drawbacks regarding material selection and application (e.g., toxicity of nanomaterials, as well as the storage and large-scale preparation of exosomes). Although the goal of all studies is to facilitate clinical application, additional work must be performed before the clinical transformation of lncRNA-targeted therapy can be achieved. To date, there have been no clinical trials on the independent use of lncRNAs as a cancer treatment. Thus, studies involving organoids and patient-derived xenografts (PDX) may accelerate this process.

## Conclusion

In summary, lncRNA molecules play a significant role in remodeling the TIME and regulating the immune escape of tumor cells. Thus, lncRNA-based targeted cancer immunotherapy has a promising future. Despite the continued problems associated with the application of lncRNA-based therapy, as research progresses and becomes optimized, the use of lncRNA as a therapeutic target will contribute to the development of novel therapeutic strategies for cancer.

## Author Contributions

B-RX, GL, and W-LJ designed the manuscript. Y-NP wrote the manuscript. Y-NP and W-CQ drew the figures and tables. B-RX, GL, and W-LJ revised the manuscript. All authors contributed to the article and approved the submitted version.

## Funding

This study was supported by the National Natural Science Foundation of China (No. 81872507), Nn10 Program of Harbin Medical University Cancer Hospital (Nn10py2017-01), and HaiYan fund of Harbin Medical University Cancer Hospital (No. JJZD2017-01) to GL; National Key Research and Development Program of China (No. 2017FYA0205302) to W-LJ; and the National Natural Science Foundation of China (No.81872430), Special Fund in China Postdoctoral Science Foundation (No. 2019T120281, 2019M661304) and Heilongjiang Province Postdoctoral Science Foundation (No. LBH-Z18109) to B-RX.

## Conflict of Interest

The authors declare that the research was conducted in the absence of any commercial or financial relationships that could be construed as a potential conflict of interest.
