# Corrigendum: Long Non-Coding RNAs in the Tumor Immune Microenvironment: Biological Properties and Therapeutic Potential

**DOI:** 10.3389/fimmu.2022.891942

**Published:** 2022-04-08

**Authors:** Ya-Nan Pi, Wen-Cai Qi, Bai-Rong Xia, Ge Lou, Wei-Lin Jin

**Affiliations:** ^1^ Department of Gynecology, Harbin Medical University Cancer Hospital, Harbin, China; ^2^ Department of Gynecology, The First Affiliated Hospital of USTC, Division of Life Sciences and Medicine, University of Science and Technology of China, Hefei, China; ^3^ Institute of Cancer Neuroscience, Medical Frontier Innovation Research Center, The First Hospital of Lanzhou University, The First Clinical Medical College of Lanzhou University, Lanzhou, China

**Keywords:** lncRNA, tumor microenvironment, immunosuppression, immune escape, therapeutic target

In the published article, there was an error regarding the affiliation details of all authors and order of affiliations, as shown below.

**Instead of the following:**

Ya-Nan Pi^1,2,^ Wen-Cai Qi^1^, Bai-Rong Xia^1*^, Ge Lou^2*^and Wei-Lin Jin^3*^

*^1^ Department of Gynecology, The First Affiliated Hospital of USTC, Division of Life Sciences and Medicine, University of Science and Technology of China, Hefei, China*

*^2^ Department of Gynecology, Harbin Medical University Cancer Hospital, Harbin, China*

*^3^ Institute of Cancer Neuroscience, Medical Frontier Innovation Research Center, The First Hospital of Lanzhou University, The First Clinical Medical College of Lanzhou University, Lanzhou, China*

**The affiliation list should read as follows:**

Ya-Nan Pi^1^, Wen-Cai Qi^2^, Bai-Rong Xia^2*^, Ge Lou^1*^and Wei-Lin Jin^3*^

*^1^ Department of Gynecology, Harbin Medical University Cancer Hospital, Harbin, China*

*^2^ Department of Gynecology, The First Affiliated Hospital of USTC, Division of Life Sciences and Medicine, University of Science and Technology of China, Hefei, China*

*^3^ Institute of Cancer Neuroscience, Medical Frontier Innovation Research Center, The First Hospital of Lanzhou University, The First Clinical Medical College of Lanzhou University, Lanzhou, China*

The authors apologize for this error and state that this does not change the scientific conclusions of the article in any way. The original article has been updated.

## Publisher’s Note

All claims expressed in this article are solely those of the authors and do not necessarily represent those of their affiliated organizations, or those of the publisher, the editors and the reviewers. Any product that may be evaluated in this article, or claim that may be made by its manufacturer, is not guaranteed or endorsed by the publisher.

